# Molecular Diagnosis of Cat Scratch Disease: a 25-Year Retrospective Comparative Analysis of Various Clinical Specimens and Different PCR Assays

**DOI:** 10.1128/spectrum.02596-21

**Published:** 2022-03-09

**Authors:** Sher Goaz, Michal Rasis, Inbal Binsky Ehrenreich, Lev Shapira, Ora Halutz, Merav Graidy-Varon, Cecilia Leibovitch, Noam Maisler, Daniel Glikman, Moshe Ephros, Michael Giladi

**Affiliations:** a The Bernard Pridan Laboratory for Molecular Biology of Infectious diseases, Tel Aviv Sourasky Medical Center, Tel Aviv, Israel; b The Azrieli Faculty of Medicine in the Galilee, Bar-Ilan University, Safed, Israel; c DYN Diagnostics, Migdal HaEmeq, Israel; d Microbiology Laboratory, Tel Aviv Sourasky Medical Center, Tel Aviv, Israel; e Infectious Disease Unit, Padeh Poriya Medical Center, Tiberias, Israel; f Pediatric Infectious Disease Unit, Carmel Medical Center and the Rappaport Faculty of Medicine, Technion-Israel Institute of Technology, Haifa, Israel; g Infectious Disease Unit, Tel Aviv Sourasky Medical Center, and The Sackler Faculty of Medicine, Tel Aviv University, Tel Aviv, Israel; University Paris-Saclay - Institute for Integrative Biology of the Cell (I2BC), CEA, CNRS

**Keywords:** *Bartonella henselae*, PCR, cat scratch disease, molecular diagnosis

## Abstract

Cat-scratch disease (CSD), caused primarily by Bartonella henselae, is a common etiology of infectious regional lymphadenopathy. Lymphadenopathy is preceded by a primary inoculation lesion and may progress to suppuration. Laboratory diagnosis of CSD is hampered by the limitations of available confirmatory tests. PCR, in general, is highly sensitive and specific; however, clinical sensitivity in CSD varies greatly between studies. We aimed to identify clinical specimens and PCR assays best suited for CSD diagnosis using a national CSD registry and a uniform case definition. Different clinical specimens and PCR assays, including conventional and real-time PCR, were evaluated. PCR was positive in 335/390 (86%) CSD patients and 425/482 (88%) PCR tests. The highest PCR sensitivity was achieved in lymph node pus aspirates (96%; *n* = 278 tests) followed by primary lesions (88%; *n* = 50), lymph node fine needle aspirations (85%; *n* = 46), lymph node biopsy specimens (73%; *n* = 91) and paraffin-embedded lymph nodes (59%; *n* = 17), (*P* < 0.001). Sensitivity was similar in all types of PCR assays studied. PCR negative predictive value of pus aspirate and lymph node biopsy specimen patient groups was 82% and 72%, respectively. Specificity was 100% based on 125 non-CSD patients with negative PCR. In conclusion, the specimen type rather than the PCR assay type has a major impact on CSD molecular diagnosis. We assume that the inadequate sensitivity of the biopsy specimens was due to sampling errors or the presence of inhibitory factors. Primary lesions should be sampled more frequently for CSD diagnosis. Physicians should be aware of the low PCR negative predictive value of lymph node biopsy specimens.

**IMPORTANCE** Polymerase chain reaction (PCR) for the detection of Bartonella henselae is an important tool for the diagnosis of cat scratch disease (CSD); however, clinical sensitivity varies greatly between studies. The current study shows that the specimen type, with pus aspiration, fine needle aspiration, and primary inoculation lesion having significantly higher sensitivity than fresh or formalin-fixed paraffin-embedded lymph node biopsy specimen, rather than the type of the PCR assay, whether a conventional or a real-time assay, has a major impact on the performance of diagnostic PCR for CSD. The new data provide new tools for the clinical microbiologist when interpreting the results of the PCR assays. Primary inoculation lesions, although easily accessible, are often neglected and should be sampled more frequently for molecular diagnosis of CSD. Physicians should be aware that negative PCR, particularly if performed on fresh or paraffin-embedded lymph node biopsy specimens, does not exclude CSD.

## INTRODUCTION

Cat-scratch disease (CSD), caused primarily by Bartonella henselae, is the most common bacterial etiology of infectious regional lymphadenopathy in adults and children. It is characterized by subacute regional lymphadenitis often associated with fever and other systemic symptoms (1–4). A typical disease course begins with a primary lesion at the site of inoculation, which develops approximately 3 to 10 days after cat or kitten contact with an inflammatory papule or a pustule that may last several weeks. Usually, 1 to 7 weeks after infection, regional lymphadenopathy occurs proximal to the primary lesion and may progress to suppuration in approximately 10% to 15% of cases. In most patients, CSD resolves spontaneously within several months (3, 4). A timely and accurate diagnosis of CSD is important not only because lymphadenitis may be painful and prolonged but also because the clinical presentation and course of CSD, usually a benign and self-limited disease, may resemble lymphoma or other malignant processes (5–7). Laboratory diagnosis of CSD, however, is still problematic owing to the limitations of available confirmatory tests. The CSD skin test is not licensed for routine use. B. henselae culture from affected lymph nodes is rarely positive. Warthin Starry silver stain is of low sensitivity and inadequate specificity. Cytology and histopathology are not specific, and an immunohistochemical assay is not available in routine diagnostic laboratories (4). Serological assays, both immunofluorescent antibody (IFA) test and enzyme immunoassay (EIA) for detection of anti–B. henselae antibodies have become the most used diagnostic tests for CSD, both as in-house and commercial products. Serology, however, has several limitations, including variable sensitivity ranging from <50% to 88%, late seroconversion, 3 weeks or more after the presentation, frequent lack of anti-*Bartonella* IgM even in documented acute infection, and cross-reactivity between antibodies to B. henselae and other *Bartonella* spp., as well as with serum of patients with Q-fever or Chlamydia pneumoniae infections (4, 8–15).

Polymerase chain reaction (PCR) is usually considered a diagnostic assay with high sensitivity and specificity and as such has been applied for the diagnosis of CSD using clinical specimens from lymph nodes, primary lesions, and other affected tissues, utilizing various approaches and methods, including real-time PCR, and several gene targets. However, the clinical sensitivity of these assays varies greatly between studies, ranging between approximately 45 and 95%, either because of poor performance of the assays, types of clinical specimens used, or lack of consensus regarding a CSD case definition (2, 16–25). This study aimed to identify clinical specimens and PCR assays best suited for molecular diagnosis of CSD using a national CSD registry and a uniform case definition.

## RESULTS

**FIG 1:**
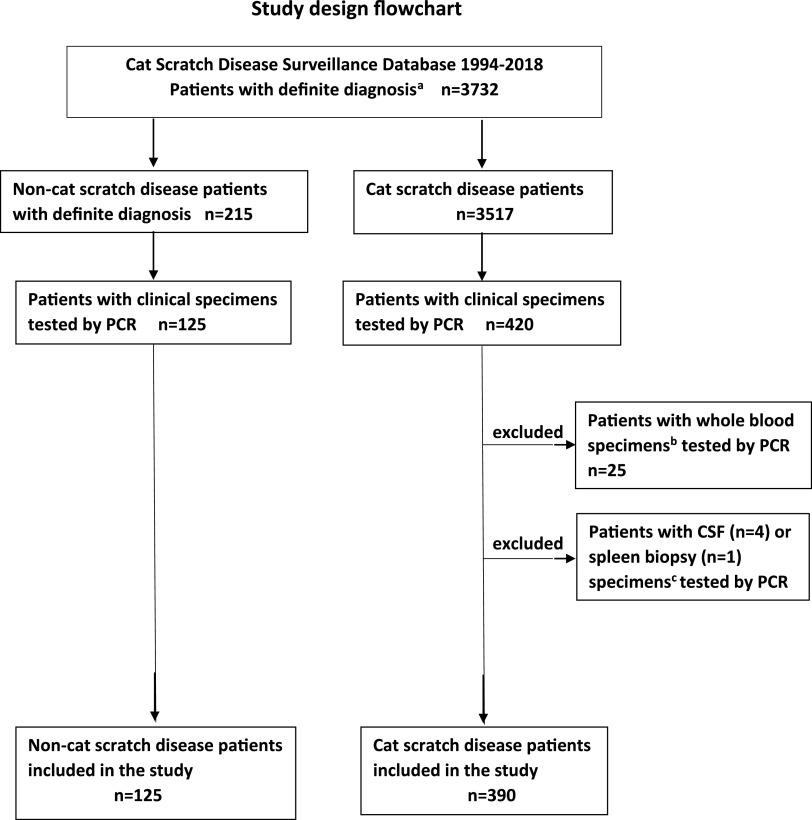
Study design flowchart. Patients with a definite diagnosis, either cat scratch disease (CSD) or non-CSD, and clinical specimens tested by PCR were identified in the CSD Surveillance Database. ^a^Cat scratch disease (CSD) surveillance database included patients with definite diagnosis, either CSD patients or patients initially suspected as having CSD but eventually receiving another definite diagnosis. ^b^Patients were excluded from the study if the only clinical specimen tested by PCR was a whole blood specimen. ^c^Patients were excluded from the study if the only specimen tested by PCR was either a cerebrospinal fluid (CSF) or a spleen biopsy specimen.

The study included 390 CSD patients with 397 PCR specimens (Fig. 1). A total of 482 PCR tests were performed, including 85 tests performed on specimens subjected to 2 or more types of PCR assays. Fifty-three specimens were tested by both conventional and real-time PCR assays.

The characteristics of the 390 CSD and the 125 non-CSD patients are presented in Table 1. The two groups were similar with regard to sex, age, the presence of fever and malaise, and the proportion of patients treated with antibiotics. Lymphadenopathy was the most frequent finding in both groups. Regional lymphadenopathy of the upper and lower limbs was significantly more common in the CSD group whereas that of the head and neck, as well as generalized lymphadenopathy, were more common in the non-CSD patient group. As expected, a history of cat/kitten contact and the presence of primary lesions were significantly more common in patients with CSD. The time from symptom onset to diagnosis was significantly shorter in the CSD patient group. Patient diagnoses of the non-CSD group are presented in Table 2.

**TABLE 1:** Characteristics of the cat scratch disease (CSD) and non-CSD patient populations

Patient characteristics	CSD patients *n* = 390	Non-CSD patients N = 125	*P* value
	n^*a*^/N^*b*^ (%)	n^*a*^/N^*b*^ (%)	
Sex-male	216/389 (56)	63/125 (50)	0.353
Age-yrs			
mean ± SD	24.4 ± 17.3	24.9 + 23.6	0.839
median (range)	22.0 (1.0-75.0)	18.0 (0.9-90.0)	0.118
Cat/kitten contact	336/390 (86)	32/125 (26)	<0.001
Lymphadenopathy	386/390 (99)	105/118 (89)	<0.001
Location of lymphadenopathy			
Axillary, epitrochlear	181/360 (50)	19/107 (18)	<0.001
Inguinal, femoral	74/360 (21)	9/107 (8)	0.004
Head and neck	108/360 (30)	60/107 (56)	<0.001
Retroperitoneum	6/360 (2)	5/107 (5)	0.137
Generalized	1/360 (0.3)	8/107 (7)	<0.001
Lymph node histopathology^*c*^			
Necrotizing granulomatous lymph	39/77 (51)	7/52 (13)	<0.001
Necrotizing or granulomatous	19/77 (25)	4/52 (8)	0.025
Lymphadenitis^*d*^			
Reactive lymphadenopathy	17/77 (22)	2/52 (4)	0.009
Fever	211/373 (57)	52/89 (58)	0.812
Malaise	176/358 (49)	38/79 (48)	0.901
Primary lesion	147/359 (41)	0/77 (0)	<0.001
Antibiotics	300/373 (80)	58/79 (73)	0.171
Time (wks) from onset to diagnosis			
mean ± SD	3.7 ± 2.7	7.2 ± 8.4	<0.001
median (range)	3.0 (1-24)	4.0 (1-52)	0.001

a n, number of patients with a specific observation.

b N, number of patients for whom data were available.

c Pathology results refer to the diagnosis of necrotizing granulomatous, granulomatous, or necrotizing lymphadenitis, or reactive lymphadenopathy.

d 11 patients had necrotizing lymphadenitis without granulomas and 8 patients had granulomatous lymphadenitis without necrosis.

**TABLE 2:** Diagnoses of patients suspected of having cat scratch disease who eventually received another definite diagnosis

Diagnosis	No. (%)
Bacterial lymphadenitis	48 (38.4)
Non-tuberculous mycobacterial lymphadenitis	19 (15.2)
Tuberculous lymphadenitis	8 (6.4)
Lymphoma (Hodgkin & non-Hodgkin)-lymphadenopathy	23 (18.4)
Carcinoma/melanoma-metastatic lymphadenopathy	9 (7.2)
Other tumors	7 (5.6)
HIV lymphadenopathy	3 (2.4)
Infected branchial cyst	3 (2.4)
Others^*a*^	5 (4.0)
Total	125 (100)

a Epidermal cyst (2 cases), Kikuchi syndrome (1 case), infectious mononucleosis (1 case), sarcoidosis (1 case).

Of the 390 CSD patients, 335 (86%) were PCR-positive. B. henselae DNA, without other *Bartonella* spp. DNA, was identified in all positive samples (Table 3). When analyzing the data in subgroups of patients by specimen type, the highest PCR sensitivity was achieved in patients with lymph node pus aspirates (95%) followed by primary lesions (86%), lymph node fine-needle aspiration (FNA) specimens (80%), lymph node biopsy specimens (69%), and formalin-fixed paraffin-embedded lymph nodes (56%). The highest negative predictive value (NPV) was recorded when testing pus aspirates (82%) followed by lymph node biopsy specimens (72%). None of the patients in the non-CSD group had a PCR-positive specimen, yielding a specificity and positive predictive value of 100%.

**TABLE 3:** Sensitivity, specificity, positive and negative predictive values of the PCR assays in cat scratch disease (CSD) and non-CSD patients categorized by specimen types

Specimen type	No. patients with positive PCR / no. patients tested		Sensitivity %	Specificity %	PPV^*a*^ %	NPV^*a*^ %
	CSD	Non-CSD				
Lymph node pus aspirate	209/221	0/55	95	100	100	82
Lymph node biopsy	51/74	0/58	69	100	100	72
Lymph node fine needle aspiration	33/41	0/2	80	NA^*b*^	NA	NA
Lymph node paraffin embedded	9/16	0/4	56	NA	NA	NA
Primary lesion	36/42	0/0	86	NA	NA	NA
Other specimens^*c*^	1/3	0/7	NA	NA	NA	NA
All cases^*d*^	335/390	0/125	86	100	100	69

a PPV, positive predictive value; NPV, negative predictive value.

b NA, not applicable. Sensitivity, specificity, PPV, and NPV were considered nonapplicable if the number of patients in each relevant specimen type category, either the CSD or the non-CSD group, was <5.

c Other specimens included 2 liver biopsy specimens and 1 spleen biopsy specimen embedded in paraffin block in the CSD group and 5 liver biopsy specimens and 2 skin biopsy specimens in the non-CSD group.

d Patients may have had more than one specimen.

**FIG 2:**
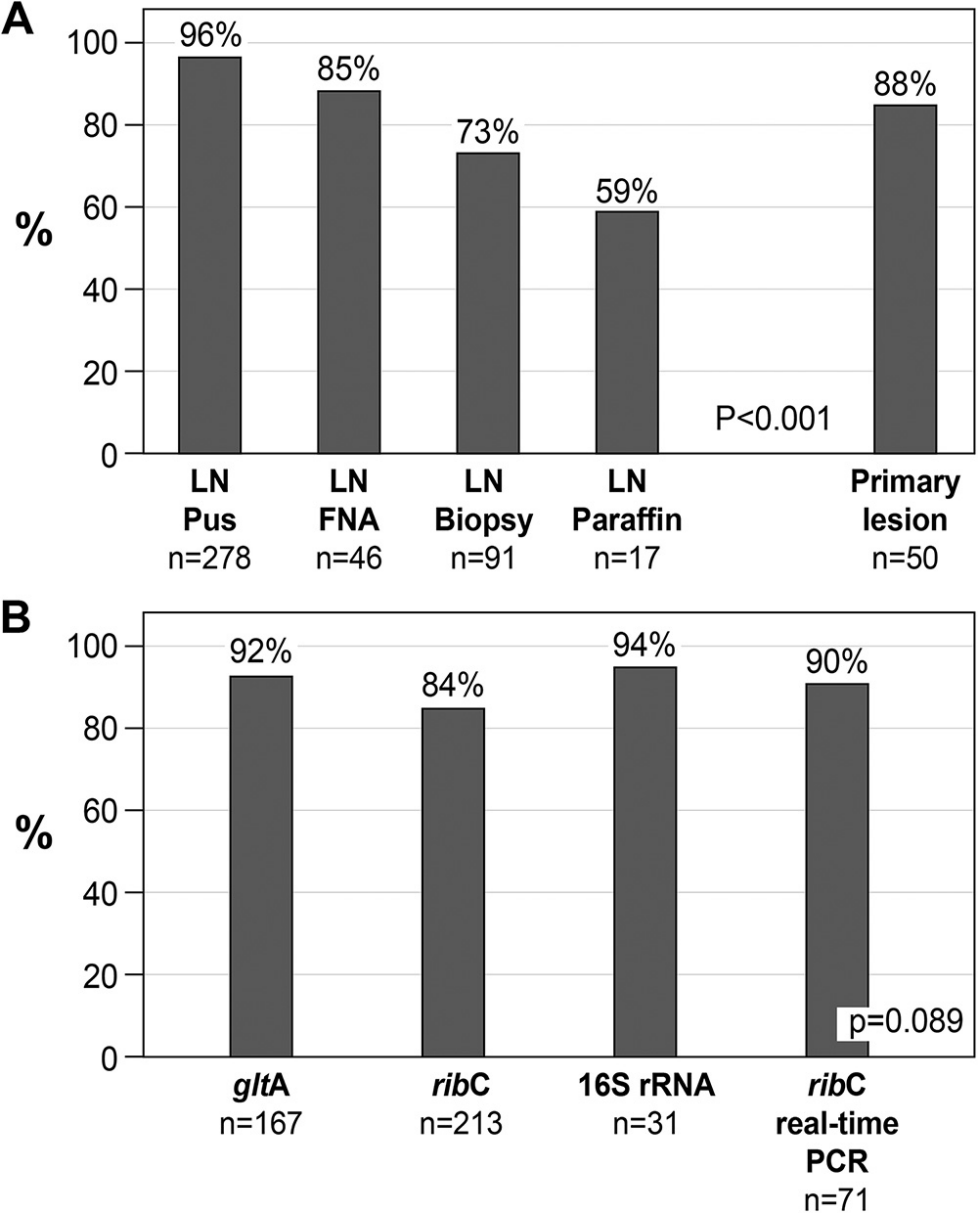
Sensitivity of PCR based on 482 tests. Numbers (*n*) and percentages are those of PCR tests performed in each category. (A) Comparison of PCR sensitivity between different types of clinical specimens. LN, lymph node; FNA, fine-needle aspiration. *P* < 0.001 for comparison of the 5 groups of clinical specimens. (B) Comparison of PCR sensitivity between different types of PCR assays. *glt*A, *rib*C, and 16S rRNA refer to the PCR target genes. *P* = 0.089 for comparison of the 4 groups of PCR assays.

The results of 482 PCR tests comparing four PCR assays and five types of clinical specimens are presented in Table 4. Similar to the patient-based analysis, the PCR test-based analysis also showed that the specimen positivity rate was significantly higher in pus aspirates (96%) followed, in decreasing order, by primary inoculation lesions (88%), lymph node FNA specimens (85%), lymph node biopsy specimen (73%), and paraffin-embedded lymph node specimens (59%), (*P* <0.001). Similar results were obtained when comparing specimen types in each of the PCR assays (*P* <0.001 to 0.032). When comparing the 4 PCR assays in each of the specimen type subgroups there was no significant difference between the PCR positivity rate regardless of the assay used (Table 4, Fig. 2).

**TABLE 4:** Sensitivity of different PCR assays categorized by specimen types

	Conventional PCR			Real-time PCR		
	gltA	ribC	16SrRNA	ribC	Total	
Target gene	n^*a*^/N^*b*^ (%)	n/N (%)	n/N (%)	n/N (%)	n/N (%)	*P* value^*c*^
Specimen type						
Lymph nodes						
Pus aspirate	102/106 (96)	105/113 (93)	24/24 (100)	35/35 (100)	266/278 (96)	0.185
Biopsy	20/25 (80)	27/41 (66)	3/5 (60)	16/20 (80)	66/91 (73)	0.463
Fine needle aspiration	20/23 (87)	13/17 (76)	2/2 (100)	4/4 (100)	39/46 (85)	0.558
Paraffin block	ND^*e*^	9/13 (69)	ND	1/4 (25)	10/17 (59)	0.250
Primary lesion	11/13 (85)	25/29 (86)	ND	8/8 (100)	44/50 (88)	0.517
Total	153/167 (92)	179/213 (84)	29/31 (94)	64/71 (90)	425/482 (88)	0.089
*P* value^*d*^	0.032	0.001	0.004	<0.001	<0.001	

a Number of positive PCR assays.

b Number of PCR assays tested.

c Comparison between types of PCR assays.

d Comparison between specimen types.

e ND, not done.

Fifty-three specimens from CSD patients (27 pus aspirates, 14 lymph node biopsy specimens, 7 primary lesions, 4 FNA, 1 paraffin-embedded lymph node) were tested by both conventional and real-time PCR. Conventional PCR was positive in 48 of 53 specimens (91%) while real-time PCR was positive in 50 of the 53 specimens (94%). There was an agreement between the 2 PCR types in 51 (96%) of the 53 specimens. The 2 discordant specimens, 1 from lymph node pus aspiration and 1 from lymph node biopsy specimen, tested positive with the real-time PCR while the conventional PCR gave false-negative results.

## DISCUSSION

The current study describes the performance of PCR assays used by a national reference center for the diagnosis of CSD. The fact that clinical specimens from CSD patients in Israel are referred for diagnosis to a single laboratory permitted analyzing the performance of these assays in many patients diagnosed with CSD during 25 years. Furthermore, utilizing a uniform CSD case definition during the entire study period, using a combination of clinical data reviewed by experts in this field and one or more positive confirmatory laboratory tests previously shown to have high specificity for CSD, increase the likelihood that these patients are true CSD cases. The EIA serological assay used in our study was previously found to be highly specific (98 to 100%), as determined by testing 3 control groups that included 340 healthy and non-CSD diseased individuals (11). PCR for B. henselae is considered to have a specificity of 100% when applied to clinical specimens of non-CSD patients, as was demonstrated in previous reports and in the current study, where all 125 non-CSD patients tested negative by PCR regardless of assay type used (16, 17).

Our study presents 2 major findings. The first is that the type of the clinical specimens is a pivotal factor when PCR clinical sensitivity is concerned. PCR positivity rate was consistently and significantly highest in lymph node pus aspirates compared with other specimens, either when analyzing the CSD patient database (Table 3) or the PCR test database in each type of the PCR assays (Table 4). PCR sensitivity of lymph node biopsy specimens, considered to be the preferable clinical specimen for diagnosis of malignant lymphadenopathy, was inferior compared with lymph node pus or FNA specimens. We believe that the most likely explanation for this observation is sampling error either because the affected lymph node is not involved in its entirety in the infectious process or because a too small, often unrepresentative fraction of the biopsied lymph node is submitted to the microbiology laboratory. We often noticed that even when an excisional biopsy specimen was performed the surgeon habitually sent a large piece for pathological evaluation while the microbiology laboratory received a tiny fragment. Our observation may also be explained hypothetically by the presence of PCR inhibitory factors in lymph node tissue that may interfere with optimal PCR assay, as suggested previously (17); however, more studies are needed to compare the effect of PCR inhibitors in pus and lymph node tissue. Prudent et al. evaluated 1762 fresh lymph node biopsy specimens of which 438 (25%) were positive for B. henselae by real-time PCR. The authors hypothesized that because they routinely received a small fragment of the entire lymph node and testing the entire lymph node may increase the diagnostic sensitivity of the PCR assay because the infection may involve only part of the lymph node (2). In contrast, necrosis with the liquefaction of infected lymph nodes due to a suppurative process usually involves an extensive portion of a lymph node and yields a relatively large amount of homogeneous pus which is more representative of the infectious process. During the FNA process, the needle is inserted and withdrawn several times in different directions and cells from multiple locations within the mass are obtained, resulting in a higher sensitivity compared with lymph node biopsy specimen. Similar to our findings, Anderson et al. (17) reported that B. henselae DNA was detected in 9 out of 9 lymph node pus aspirates (100%) but only in 12 of 16 (75%) lymph node biopsy specimens. Also, Bergmans et al. (20) reported that of 89 pus aspirates from skin test-positive patients collected over 8 years, 85 (96%) were PCR positive while 82 of 137 (60%) lymph node-derived clinical samples, pus aspirates, and biopsy specimens, obtained from patients with clinically suspected CSD tested positive by PCR. However, the actual number of biopsy specimens and pus aspirates in the latter group was not provided (20). In another study B. henselae DNA was detected in one out of 18 lymph node biopsy specimens and in 3 of 3 lymph node aspirates obtained from patients with possible CSD, using conventional PCR with an analytical sensitivity of 10^−4 ^ng or 50 genome copies (26). Contrary to these findings, in a recent study from Italy 56 pus aspirates and 39 fresh lymph node biopsy specimens were tested by real-time PCR and found to have similar B. henselae detection rates of 38% and 36%, respectively. An explanation for the low detection rate, particularly in pus specimens, was not discussed (27). In a previous study of 27 children with mycobacterial lymphadenitis from whom both FNA and lymph node tissue specimens were obtained, real-time PCR for Mycobacterium spp. yielded significantly more positive results for FNA (25/27 [93%]) than for tissue biopsy specimen (16/27 [59%]; *P* = 0.003) (28). Similarly, our study also suggests that due to the better diagnostic yield and the less-invasive nature of FNA in comparison to biopsy specimen, a physician should consider FNA as the preferred diagnostic procedure when facing lymphadenopathy before suppuration if CSD is highly suspected. Other studies either tested only lymph node biopsy specimens (1, 29) or did not provide sufficient data for a comparative evaluation of PCR performance on lymph node biopsy specimen versus pus aspirate specimens (16, 21–23, 25, 30). Physicians should be aware that primary inoculation lesions are important specimens for molecular diagnosis of CSD, particularly when lymph node specimens, especially pus, are not available, as shown in our study. Carithers et al. (31), in a detailed clinical study of 1200 CSD patients, stated that locating the primary lesions is the most neglected feature in the study of patients with CSD and reported that meticulous physical examination could identify such lesions in 93% of patients. Formalin-fixed paraffin-embedded lymph node biopsy specimens had the lowest sensitivity, and, although we processed only 17 specimens, we believe that the low sensitivity reflects the true performance of PCR in these specimens because it agrees with previous studies (18, 32). It has been suggested that the low recovery rate of B. henselae DNA is likely to be due to the various steps of fixation and embedding of the tissues and deparaffinization, known to damage DNA (18).

Our second finding is the lack of significant difference between the performance of the various types of PCR assays, including real-time PCR, used during the study period. Real-time PCR, in addition to its more rapid test cycle turnaround time, its amenability to automation, and the decreased risk of false-positive results due to DNA carryover, is notable for its higher sensitivity compared with conventional PCR (33). In the current study, among 53 CSD specimens that were tested by both conventional and real-time PCR, we identified only 2 specimens (4%) that were positive by real-time PCR and negative by conventional PCR. The overall clinical sensitivity of real-time PCR (90%) was not significantly different compared to conventional PCR assays, despite a high analytical sensitivity of 3 to 30 copies per reaction. We assume that the bacterial load in most of the PCR-positive CSD clinical specimens tested was high enough to be detected by conventional PCR assays so that the real-time PCR did not significantly increase the clinical sensitivity. Similarly, in a previous study, 73 clinical samples, mainly pus aspirates, from patients suspected of having CSD were tested by both conventional and real-time PCR and 29 (40%) were positive with 100% agreement between the two assays. The lower detection limit of the real-time PCR assay was 10 to 100 fg of DNA, indicating high analytical sensitivity. The clinical sensitivity of these assays was difficult to evaluate due to insufficient clinical data (24).

Our study has several limitations mainly due to its retrospective nature. First, head-to-head comparison of the same specimens using different PCR methodologies and target genes was not applicable for most of the clinical samples submitted to the laboratory over 25 years because many of these specimens were not available for such a comparison. Second, data were incomplete regarding the size, weight, and the fraction of the excised lymph node out of the entire node, making the analysis of PCR results of lymph node biopsy specimens, shown to be inferior to pus samples, more difficult. Third, to ensure that only CSD patients are included in the database, patients were excluded from the study if clinical information essential for supporting the diagnosis of CSD was missing. This approach could have hypothetically resulted in the exclusion of non-CSD patients with false-positive laboratory results.

It should also be emphasized that despite the superb specificity and relatively high sensitivity, depending on the specimen type, PCR testing requires tissue samples not always available for CSD diagnosis. Blood specimens, although readily available, have unacceptably low PCR sensitivity for the diagnosis of CSD (34). Serology, therefore, with all its limitations, remains the main diagnostic modality of CSD.

In conclusion, the specimen type rather than the type of the PCR assay has a major impact on the performance of molecular diagnosis of CSD. While real-time PCR is the diagnostic assay of choice due to its many advantages, our study demonstrates that its higher analytical sensitivity does not always translate into a higher detection rate of B. henselae DNA from clinical CSD specimens. Although pus aspirates from affected lymph nodes have the highest recovery rate of B. henselae DNA, suppuration, unfortunately, occurs only at a late stage of CSD lymphadenitis and occurs in no more than one-sixth of patients (4). Primary inoculation lesions, although easily accessible, are often neglected and should be sampled more frequently for molecular diagnosis of CSD. Physicians should be aware that negative PCR, particularly if performed on fresh or paraffin-embedded lymph node biopsy specimens, does not exclude CSD.

## MATERIALS AND METHODS

### CSD surveillance study and patient population.

A surveillance study of CSD has been conducted in Israel since 1991. Clinical specimens for the detection of B. henselae infection, including serology, PCR assays, and cultures obtained from patients suspected to have CSD, are sent to a single laboratory at Tel Aviv Sourasky Medical Center. Demographic, epidemiologic, and clinical data are collected using a structured questionnaire sent to the referring physician. Missing information is obtained by contacting the referring physician, the patient, or the patient’s family. Medical records of hospitalized patients are reviewed and histopathologic, cytologic, microbiologic and imaging reports are obtained. Data are recorded in the CSD Surveillance Database. For the surveillance study, a case of CSD was defined as a patient meeting the following criteria. (i) A clinical syndrome consistent with CSD in the absence of another diagnosis; (ii) at least one confirmatory laboratory result: positive serology for anti-B. henselae antibodies (IgM and/or IgG), and/or positive PCR for B. henselae DNA, and/or positive B. henselae culture. History of cat contact is considered supportive evidence and not a prerequisite for the case definition. The study was approved by the institutional review board (Helsinki committee) of the Tel Aviv Sourasky Medical Center.

### Study design.

CSD cases with documented PCR assays were identified in the CSD Surveillance Database of the years 1994 to 2018. A final diagnosis of CSD was made only after a review of each case by the principal investigator (M.G.). In uncertain cases, another investigator (M.E.) was consulted, and the final diagnosis was determined by agreement. Five patients with positive PCR results but without sufficient clinical data to support/exclude a diagnosis of CSD were excluded from the CSD Surveillance Database. Patients for whom only whole blood (*n* = 25; all negative), cerebrospinal fluid (*n* = 4, all negative), or spleen biopsy (*n* = 1, negative) specimens were available for PCR were excluded from the study (Fig. 1). To determine the specificity of the PCR assays, we analyzed PCR results of patients whose specimens were referred to our laboratory because they were suspected to have CSD but were eventually found to have other definite diagnoses (*n* = 125). Similar to the patients in the CSD group, attempts were made to obtain demographic, epidemiologic, and clinical data for these patients.

### Clinical specimens.

The following clinical specimens were tested by PCR. Lymph node biopsy specimens were obtained by open or core needle procedures. Fine-needle aspiration (FNA) of lymph nodes smeared onto glass microscope slides which were unstained and air-dried, as previously described (35), or placed into 1.5-mL microtubes. Pus aspirated from suppurated lymph nodes. Sections (10 μm thick) of formalin-fixed paraffin-embedded lymph node tissue. Material extracted from primary inoculation lesions obtained as previously described (35) or skin punch biopsy specimens of primary inoculation lesions.

### DNA extraction and PCR assays.

DNA from clinical specimens was extracted as previously described utilizing various protocols (19, 35, 36) or using the QIAamp DNA Minikit (Qiagen, Valencia, CA.). Different PCR assays using various protocols and targeting different genes were used during the 25-year study period. Conventional PCR assays targeting the *glt*A gene with restriction fragment length polymorphism (RFLP) analysis of the PCR products, or the *rib*C gene with either RFLP analysis or additional PCR assay targeting the *pap*31 gene were performed as previously described (19, 22, 26, 36). Conventional PCR assay using the broad host range approach targeting the 16S rRNA gene with either RFLP analysis, DNA sequencing, and/or dot blot hybridization of the PCR products was performed as previously described (19, 35, 37, 38). Conventional PCR assay targeting the *htr*A gene, used in our lab in 21 CSD clinical specimens from 1994 to 1996, was excluded from the analysis due to unacceptably low sensitivity and inadequate specificity (19, 39), and all 21 specimens were retested with another PCR assay. Real-time PCR for *Bartonella* spp. was developed in our research laboratory and introduced into the clinical microbiology laboratory in 2016. The CSD assay was designed to specifically identify B. henselae and identify, at the genus level, other *Bartonella* spp. potentially implicated in CSD. Detailed descriptions and principles of the assay have been recently reported (40). Briefly, *Bartonella* genus-specific degenerate primers were used to amplify a 185 bp fragment of the *rib*C gene. The resulting PCR product was detected postamplification using 2 SimpleProbe probes (Roche Diagnostics, Basel, Switzerland) for the identification of *Bartonella* genus and B. henselae DNA (TIB Molbiol, Berlin, Germany, and Dyn Diagnostics, Migdal HaEmeq, Israel). Each probe has a distinctive melting temperature in which the probe detached from the amplified target, resulting in a rapid drop in the fluorescent signal. Real-time PCR and analysis were performed using the LightCycler 96 instrument and software version 1.1. (Roche Diagnostics, Basel, Switzerland). For positive controls, 30 and 3000 copies of pMR-ribC185-Bh, a plasmid containing the *rib*C PCR amplicon of B. henselae, were used along with clinical specimen DNA in a multiplex real-time PCR assay. The analytical sensitivity was determined to be 3 to 30 plasmid copies per reaction (Giladi M, unpublished data). To identify *Bartonella* spp., melting peaks of unknown specimens were compared to those of known standard (positive-control plasmid). Each run of clinical specimens also included a negative no-template control and an internal control gene (human *β-globin*) to verify template DNA integrity and the absence of PCR inhibitors in the specimens.

### Laboratory tests.

EIA for detection of anti–B. henselae antibodies were performed and interpreted as reported previously (11, 41, 42).

### Statistical analysis.

Data regarding PCR performance were analyzed separately in the CSD patient group (*n* = 390) and the PCR tests group (*n* = 482). Categorical variables were compared using the chi-square test or Fisher's exact test and continuous variables were compared using the *t* test or Mann-Whitney test. All statistical tests were two-tailed. *P* < 0.05 was considered significant. Statistical analyses were performed using SPSS software, version 22.0 (Chicago, IL).
